# Psychometric properties of patient-reported outcome measures (PROMs) in wrist osteoarthritis: test–retest reliability and construct validity

**DOI:** 10.1186/s12891-022-05511-6

**Published:** 2022-06-09

**Authors:** Sara L. Larsson, Elisabeth Brogren, Lars B. Dahlin, Anders Björkman, Elisabeth Ekstrand

**Affiliations:** 1 Department of Hand Surgery, Skåne University Hospital, Jan Waldenströms gata 5, Malmö, SE-205 02 Sweden; 2 Department of Translational Medicine - Hand Surgery, Skåne University Hospital, Jan Waldenströms gata 5, 205 03 Malmö, Sweden; 3 Department of Biomedical and Clinical Sciences, Linköping University, Linköping, Sweden; 4 Department of Hand Surgery, Institute of Clinical Sciences, Sahlgrenska University Hospital, Sahlgrenska Academy, University of Gothenburg, Gothenburg, Sweden; 5 Department of Health Sciences, Lund University, Lund, Sweden

**Keywords:** Wrist osteoarthritis, Patient-reported outcome measures, Psychometric properties, NRS, DASH, PRWE

## Abstract

**Background:**

Patient-reported outcome measures (PROMs) are frequently used to assess the effects of treatments in patients with wrist osteoarthritis (OA), but their psychometric properties have not been evaluated in this group of patients. Our aim was to evaluate the psychometric properties of the Numeric Rating Scale (NRS pain at rest, pain on motion without load, and pain on load), the Disabilities of the Arm, Shoulder and Hand (DASH) and the Patient Rated Wrist Evaluation (PRWE) questionnaires in patients with wrist OA regarding test–retest reliability and construct validity.

**Methods:**

The NRS, DASH and PRWE were self-administered by 50 patients (40 men and 10 women, mean age 66 years) in a postal survey on two occasions, two weeks apart. Test–retest reliability was evaluated by Kappa statistics and the Spearman rank correlation coefficients (rho) were calculated to evaluate construct validity.

**Results:**

The Kappa coefficients for DASH, PRWE and NRS pain on motion without load and NRS pain on load were > 0.90, 95% CI ranging from 0.84 to 0.98, while NRS pain at rest was 0.83, 95% CI 0.73–0.92. The construct validity of the PROMs was confirmed by three formulated hypotheses: a higher correlation between PRWE and NRS (rho 0.80–0.91, *p* < 0.001) was found, compared to DASH and NRS (rho 0.68–0.80, *p* < 0.001); the NRS pain on motion without load and NRS pain on load correlated more strongly to PRWE and DASH (rho 0.71–0.91, *p* < 0.001) compared to NRS pain at rest (rho 0.68–0.80) and a high correlation between PRWE and DASH was found (rho 0.86, *p* < 0.001).

**Conclusions:**

The NRS, DASH and PRWE demonstrate excellent test–retest reliability and moderate to high construct validity in patients with wrist OA. These PROMs are highly related, but they also differ. Therefore, they complement each other in ensuring a comprehensive evaluation of perceived disability in wrist OA. As PRWE showed the highest test–retest reliability and the highest relation to the other PROMs, the sole use of the PRWE can be recommended in clinical practice.

## Background

Patients with wrist osteoarthritis (OA) commonly suffers from pain, decreased range of motion (ROM) and reduced grip strength [1, 2]. These impairments can have a significant impact on patient’s ability to perform daily activities and to participate in society [3]. Reducing pain and improving the patient’s ability to use their hand and wrist in daily activities are a prime focus in non-surgical and surgical treatment strategies for patients with wrist OA [1, 2, 4].

Indications for interventions in patients with wrist OA are often the patient’s self- perceived symptoms and disabilities [3]. Therefore, patient-reported outcome measures (PROMs) are important to include when assessing the effects of different treatments for wrist OA because they provide a more complete picture of the disability from the patient’s perspective [5, 6].

In wrist and hand OA, commonly used PROMs are pain rating scales, such as the Visual analogue Scale (VAS) and the Numerical Rating Scale (NRS) [7–10] for measuring pain intensity, the Disabilities of the Arm, Shoulder and Hand (DASH) questionnaire [8, 11–13], and the Patient-Rated Wrist Evaluation (PRWE) questionnaire [14, 15]. The DASH measures self-reported upper extremity physical function and symptoms taking the whole upper extremity into account, irrespective of which hand or if both hands are used and is the most commonly used PROM in clinical trials for patients with wrist OA [8]. The PRWE is a wrist specific PROM originally developed for the assessment of perceived disability after a distal radius fracture. Content validity of PRWE has previously been evaluated in the context of hand and wrist arthritis (27% with OA, 67% with rheumatoid arthritis and 6% with psoriatic arthritis) [16], but apart from this, none of these PROMs have been evaluated regarding their psychometric properties in the context of wrist OA.

As the psychometric properties of a measure are closely linked to the population it is intended for, there is a need to investigate the psychometric properties for these PROMs in patients with wrist OA [17, 18]. The aim of our study was to assess and compare the psychometric properties of the NRS, DASH, and PRWE in a group of patients with wrist OA regarding test–retest reliability and construct validity.

## Material and methods

### Participants

Data were collected from January 2020 to December 2021. Inclusion criteria were 1) radiographically confirmed wrist OA and 2) age ≥ 18. Exclusion criteria were 1) the presence of other diseases or disorders that could affect arm and hand function, 2) previous surgery to the wrist, and 3) inability to understand and follow test instructions due to communicative, mental, or cognitive impairments.

Via the hospital’s administrative patient system, 66 patients (54 men and 12 women) seeking medical care for wrist OA at the Department of Hand Surgery, Skåne University Hospital Malmö, Sweden between the years 2016 to 2021 were identified by reviewing the patients’ medical records. Thirteen patients did not respond and three declined to participate, leaving 50 patients that were included in the study.

Prior to inclusion, information about the purpose of the study was provided, and all participants gave their written consent to participate. The principles of the Declaration of Helsinki were followed. The study was approved by the Swedish Ethical Review Authority, Dnr 2019–02,437. The COnsensus-based Standards for the selection of health Measurement INstruments (COSMIN) checklist, guidelines for methodological quality in psychometric studies, was followed [19].

### Outcome measures

The NRS [20–22] is a numeric 11-point pain rating box scale with numerical descriptors on the box, ranging from 0 representing one pain extreme (no pain) to 10 representing the other pain extreme (worst pain imaginable). Patients select a value that is most in line with the intensity of pain they have perceived in the affected wrist over the last week. Three measures of pain are rated in this study: 1) pain at rest, 2) pain on motion without load, and 3) pain on load [23]. The NRS have been found to be valid and reliable in different acute and chronic pain conditions and in healthy populations [20, 22].

The main part of the DASH is a 30-item disability/symptom scale concerning the patient's health status during the preceding week [11, 12]. The items ask about the degree of difficulty in performing different physical activities because of arm, shoulder, or hand problems (21 items), the severity of each of the symptoms of pain, activity-related pain, tingling, weakness and stiffness (5 items), as well as the problem's impact on social activities, work, sleep, and self-image (4 items). Each item has five response options. The scores for all items are then used to calculate a scale score ranging from 0 (no disability) to 100 (most severe disability). The score for the disability/symptom scale is called the DASH score. The DASH is one of the most important PROMs for measuring upper extremity disability, and its psychometric properties have been evaluated in a range of conditions involving the upper extremity [24, 25]. In this study, the validated Swedish version of DASH was used [13].

The PRWE [14] includes 15 questions, divided into two subscales assessing pain (5 items) and function (10 items, 6 concerning specific tasks and 4 the ability to perform daily activities) over the past week. The questions are scored on a 10-point ordered categorical scale, ranging from no pain or no difficulty (0 points), to worst pain or unable to do (10 points). The total score of the subscales pain (sum of 5 items) and function (sum of 10 items divided by 2) ranges from 0 to 50. The maximum total score of PRWE is 100 and represents the worst disability, whereas 0 represents no disability. The PRWE has mainly been found to be valid and reliable in patients with distal radius fractures, but also in a variety of other hand and wrist-related injuries and disorders [7]. In the current study, the validated Swedish version of PRWE by Wilcke et al. was used [26].

The quality of a PROM depends on its psychometric properties, such as reliability and validity [18]. Reliability implies that the measure produces similar results under consistent conditions. We evaluated this in a test–retest situation, i.e. the outcome measures should ideally produce similar results from one test occasion to the next, thus indicating that the measurements are stable over time [17]. Validity refers to the degree of which a test measures what it is intended to measure, and can be evaluated as construct validity, where outcomes of the same construct should be related to each other [18]. As no ´gold standard´ exists for PROMs, the commonly used way to investigate construct validity is to test hypotheses about expected relationships with other outcome measures of good quality [27]. To assess construct validity, we have chosen three reliable and valid PROMs frequently used to evaluate the effect of treatment on patients with wrist OA. We formulated the following hypotheses: 1) PRWE should correlate more strongly to NRS compared to DASH and NRS because PRWE includes the subscale pain and both NRS and PRWE are wrist specific; 2) NRS pain on motion without load and NRS pain on load should correlate more strongly to PRWE and DASH compared to NRS pain at rest as both PRWE and DASH mainly contain activity based questions; and 3) PRWE and DASH should have a moderate to high correlation since they measure the same construct, i.e. upper extremity disability. However, since these questionnaires differ slightly, with DASH being more generic than PRWE, we did not expect a very high correlation (> 0.90).

### Procedures

Information about the study with an informed consent form was sent to the participants by surface mail together with the PROMs (NRS, DASH and PRWE) for test occasion 1 (T1). The participants noted the date when the questionnaires were responded and returned them in a prepaid envelope. When the researcher had received the responses of the T1 questionnaires, the same questionnaires for test occasion 2 (T2) were sent to the participants. If a participant failed to send T2 within two weeks, he/she was reminded by telephone or a second surface mail. The time interval of the responses between T1 and T2 became approximately two weeks, with some outliers taking longer time, which is standard in test–retest studies [18, 28].

Background data – such as gender, age, affected side and handedness – were obtained from the medical records. All patients included in the study had wrist radiographs and/or computer tomography taken prior to referral to our clinic. The radiographs were evaluated by an experienced hand surgeon (EB), and the type of wrist OA (scapholunate advanced collapse; SLAC, scaphoid non-union advanced collapse; SNAC, idiopathic wrist OA or Mb Kienböck) was recorded (see Table 1). If the participants had bilateral wrist OA, the wrist that the participant reported as the most affected was included in the wrist specific assessments.

**Table 1:** Characteristics of 50 participants with wrist osteoarthritis (OA)

Characteristics	
Age, mean (SD; min–max)	66 (9; 41–79)
Male sex, n (%)	40 (80)
Affected wrist, dominant, n (%)	33 (66)
Type of OA, n (%)	
SLAC	38 (76)
SNAC	6 (12)
Idiopathic OA	5 (10)
Mb Kienböck	1 (2)
Days between T1-T2, mean (SD; min–max)	16.7 (17.4; 4–86)

*SLAC* Scaphoid Lunate Advanced Collapse, *SNAC* Scaphoid Non-union Advanced Collapse, *T1* Test occasion 1, *T2* Test occasion 2

### Statistical analysis

Descriptive statistics – such as means, standard deviations (SD), frequencies, median (range) – were calculated as appropriate.

The test–retest reliability of NRS (pain at rest, pain on motion without load, and pain on load), DASH and PRWE (total score and subscales pain and function) were evaluated by Kappa statistics (the proportion of agreement observed beyond the agreement expected by chance) using quadratic weights [28–30]. The strength of the Kappa coefficient can be interpreted as follows: < 0.40 poor, 0.40 to 0.75 fair to good, and > 0.75 excellent [31].

The Spearman rank correlation coefficients (rho) were calculated to evaluate construct validity. The PRWE and DASH were correlated to NRS, and the correlation between PRWE and DASH was also calculated. Data from the first test occasion were used in the construct validity analyses. The strength of the correlations was interpreted as follows: rho < 0.5 low, 0.5 to < 0.7 moderate, > 0.7 high [32].

Data were analyzed with the IBM SPSS Statistics version 27 (IBM Corporation, Armonk, New York, United States). *P*-values < 0.05 were considered statistically significant.

## Results

The clinical characteristics of the 50 participants are presented in Table 1. Their mean age was 66 years (SD 9) and the dominant hand was the most commonly affected. A majority of the participants were diagnosed with a SLAC wrist. The mean interval between the responses was 16.7 days (SD 17).

The Kappa coefficients for DASH, PRWE (total score and the subscales pain and function) and NRS pain on motion without load and NRS pain on load were > 0.90, 95% CI ranging from 0.84 to 0.98, while NRS pain at rest was 0.83, 95% CI 0.73–0.92 (Table 2).

**Table 2:** Test–retest reliability of the NRS, DASH and PRWE in 50 patients with wrist osteoarthritis (OA)

PROMs	T1, median (IQR)	T2, median (IQR)	Kappa coefficient (CI 95%)
NRS			
Pain at rest	3 (1–5)	3 (1–6)	0.83 (0.73–0.92)
Pain on motion without load	6 (3–8)	6 (3–8)	0.91 (0.86–0.95)
Pain on load	8 (5–9)	7 (5–9)	0.92 (0.87–0.96)
DASH	37.1 (21.5–55.4)	38.4 (22.9–54.6)	0.91 (0.84–0.98)
PRWE			
Subscale Pain	30.5 (22.5–38.0)	31.5 (23.0–38.3)	0.93 (0.90–0.97)
Subscale Function	26.8 (13.4–34.7)	24.5 (14.7–36.0)	0.93 (0.88–0.98)
Total score	56.0 (31.5–69.5)	53.5 (35.4–75.4)	0.94 (0.91–0.98)

*T1* Test occasion 1, *T2* Test occasion 2, *IQR* Interquartile range, *CI* Confidence interval, *NRS* Numeric Rating Scale (score range 0–10), *DASH* Disabilities of the Arm, Shoulder and Hand (score range 0–100), *PRWE* Patient-Rated Wrist Evaluation (subscale pain score range 0–50, subscale function score range 0–50, total score range 0–100)

The highest correlations were found between PRWE (total score and subscales) and NRS pain on motion without load (rho 0.89–0.91, *p* < 0.001), and between PRWE total score and DASH (rho 0.86, *p* < 0.001). The PRWE subscale pain correlated higher (rho 0.84, *p* < 0.001) than the other measures to NRS pain at rest. Somewhat lower correlations were seen between DASH and NRS (rho 0.68–0.80, *p* < 0.001) (Table 3).

**Table 3:** Spearman rank correlations (rho) between NRS, DASH and PRWE in 50 patients with wrist osteoarthritis (OA)

PROMs	PRWE total		PRWE pain		PRWE function		DASH	
	**Rho**	***P-*value**	**Rho**	***P-*value**	**Rho**	***P-*value**	**Rho**	***P-*value**
NRS pain at rest	0.80	< 0.001	0.84	< 0.001	0.74	< 0.001	0.68	< 0.001
NRS pain on motion without load	0.91	< 0.001	0.89	< 0.001	0.89	< 0.001	0.80	< 0.001
NRS pain on load	0.86	< 0.001	0.79	< 0.001	0.80	< 0.001	0.71	< 0.001
DASH	0.86	< 0.001	0.83	< 0.001	0.84	< 0.001	-	-

*NRS* Numeric Rating Scale (score range 0–10), *DASH* Disabilities of the Arm, Shoulder and Hand (score range 0–100), *PRWE* Patient-Rated Wrist Evaluation (total score range 0–100)

## Discussion

This study has evaluated test–retest reliability and construct validity for the NRS, DASH, and PRWE on patients with wrist OA. Our results showed excellent test–retest reliability for these PROMs when applied to this specific group of patients. High correlations were seen between PRWE, NRS and DASH, whilst moderate to high correlations were seen between DASH and NRS.

The NRS, DASH and PRWE are rated in ordinal scales, therefore, retest reliability was analysed with Kappa statistics as recommended [27]. In a systematic review by Shafiee et al. [7], the intraclass correlation coefficient (ICC) was included when evaluating test–retest reliability, which should actually be used when evaluating agreement for parametric data [33]. Thus, the retest reliability results of the current study are not fully comparable even though a quadratic kappa gives about the same result as an ICC [34].

Excellent agreement according to the kappa-coefficient was found for the NRS (0.83–0.92). Earlier studies have mainly used a visual analogue scale (VAS) score evaluating general pain intensity for the wrist, but also with excellent test–retest agreement (ICC 0.84) [7, 35]. Although high test–retest agreement of NRS has been reported [36], it is not as thoroughly investigated as VAS [22]. A high correlation between VAS and NRS has been established, and NRS is recommended to use when assessing pain intensity on the basis of higher compliance rates, better responsiveness, ease of use, and good applicability relative to VAS [20]. Our study showed that NRS pain at rest had somewhat lower Kappa coefficient (0.83) compared to NRS pain on motion without load and NRS pain on load (0.91–0.92). Osteoarthritic pain is complex and variable with “good days and bad days” mainly occurring on use, movement, weight-bearing and later in the day [3, 37]. All these factors probably affect pain at rest, thereby resulting in more pain variations.

An excellent kappa coefficient was found for DASH in our study (0.91). This is in line with previous studies [13, 38–42] that have shown excellent test–retest agreement for DASH with ICC > 0.90 in patients with various upper extremity disorders. The PRWE also showed excellent agreement for the total score and the subscales (Kappa coefficients 0.93–0.94). This is in accordance with a previous systematic review evaluating test–retest reliability for PRWE in 24 studies of various types of wrist and hand injuries [7] that reported ICCs of 0.91–0.93 [7]. Taken together, the NRS, DASH and PRWE can be considered reliable PROMs in wrist OA; thus, they produce similar results under consistent conditions.

When construct validity was evaluated, the formulated hypotheses were confirmed. The PRWE correlated higher with the NRS pain scale compared to DASH. Since PRWE contains a very detailed section with five specific pain questions compared to DASH, which only contains two general pain questions, this was a rather expected result. Previous studies, including patients with various shoulder-, hand/wrist disorders as well as RA patients with upper extremity disabilities, have mainly found moderate to high correlations between DASH and VAS (rho 0.60–0.72) [38, 39, 41, 42]. However, one study, including various arm, shoulder and hand problems, [40] demonstrated low to moderate correlations between DASH and VAS pain at rest (rho 0.44) and VAS pain during activity (rho 0.56). Possible explanations for this low correlation could be that the recruited subjects had heterogeneous diagnoses with variable degrees of pain intensity. In accordance with the present study, Beaton et al. [38] found a high correlation (> 0.70) between DASH and VAS in patients with various shoulder- and hand/wrist disorders. Moderate to high correlations have also been found between PRWE and VAS (rho 0.50–0.74) [43–46]. When PRWE and DASH were correlated to NRS, the highest correlations were found for NRS pain on motion without load. This can be explained by the fact that most questions in both PRWE and DASH concern the disability on activities of daily living.

High correlations were found between DASH and PRWE (rho 0.86), which is comparable to previous studies that have found moderate to high correlations between DASH and PRWE (rho 0.61–0.94) [7]. Our results are expected since DASH and PRWE to a large extent resemble each other in rating the same construct, i.e., upper extremity disability, predominantly in activities. However, even if they are highly correlated, they also differ slightly probably as DASH is a generic upper limb instrument, whereas PRWE is wrist specific.

The application of self-reported outcome measures allows healthcare professionals to assess the course of treatment; furthermore, it facilitates comparison between groups in clinical trials [8]. To include different types of PROMs – such as symptom specific, wrist specific and more generic questionnaires – provides the opportunity to evaluate separate symptoms and overall self-reported upper extremity disability. This strengthens the concept that NRS, DASH and PRWE complement each other when evaluating patients with wrist OA, especially in a research setting. However, in clinical practice, barriers exists in incorporating PROMs to measure outcomes into the clinical routine because such measurements are time-consuming; thus, they can decrease productivity [47]. As we found a high correlation between PRWE and both DASH and NRS, all three PROMs may not be necessary for the assessment of wrist OA. The PRWE has been shown to be easier for patients to complete, quicker to administer and easier to score than DASH [47]. Consequently, PRWE might be recommended to be used as a sole PROM in clinical practice for assessing functional outcome of wrist OA.

### Strengths and limitations

One of the main strengths of our study is the investigation of test–retest reliability and construct validity for a specific patient group, wrist OA, since a measure’s psychometric properties are closely linked to the population they are intended for [17, 18].

According to the COSMIN checklist, the number of participants in this study (*n* = 50) is an adequate sample size. However, a sample size ≥ 100 would have been considered as very good [18, 48]. The time interval between the repeated ratings in our study is approximately two weeks, which is recommended [18]. However, as there were a few participants that took longer than two weeks to respond to T2, they needed to be reminded, which can be seen as a minor limitation. None of the participants were actively seeking care for their wrist problems during the data collection since data was collected retrospectively, but we cannot fully ensure that the patients were completely stable in the interim period. Most patients were men (80%), and SLAC wrist was the most represented diagnosis (76%). This is characteristic of patients with wrist OA [4, 49], but it can limit the generalization to the whole wrist OA population.

This study provides novel data on the psychometric properties of three important PROMs used to assess perceived symptoms and disabilities in patients with wrist OA, which can improve the evaluation of different treatments for this group of patients. The recommended sole use of PRWE could also increase the use of PROMs in clinical practice. For future research, it would also be valuable to evaluate the responsiveness of these PROMs in patients with wrist OA.

## Conclusions

The NRS, DASH and PRWE demonstrate excellent test–retest reliability and moderate to high construct validity in patients with wrist OA. These PROMs are highly related, but they also differ. Therefore, they complement each other in ensuring a comprehensive evaluation of perceived disability in wrist OA. As PRWE showed the highest test–retest reliability and the highest relation to the other PROMs, the sole use of the PRWE can be recommended in clinical practice.

## Data Availability

The datasets generated and/or analysed during the current study are not publicly available but available from corresponding author on reasonable request. Public access to data is restricted by the Swedish government (Public Access to Information and Secrecy Act; https://www.government.se/information-material/2009/09/public-access-to-information-and-secrecy-act/). However, data may be available for researchers upon special review and includes approval of the research project by both an Ethics Committee at national level and governmental data safety committees.

## Abbreviations

OA: Osteoarthritis
ROM: Range of Motion
PROM: Patient-Reported Outcome Measure
NRS: Numeric Rating Scale
VAS: Visual Analogue Scale
DASH: Disabilities of the Arm, Shoulder and Hand
PRWE: Patient-Rated Wrist Evaluation
CMC: Carpometacarpal
T1: Test occasion 1
T2: Test occasion 2
COSMIN: COnsensus-based Standards for the selection of health Measurement INstruments
SLAC: Scapholunate Advanced Collapse
SNAC: Scaphoid Non-union Advanced Collapse
ICC: Intraclass Correlation Coefficient
IQR: Interquartile Range
CI: Confidence Interval
SD: Standard Deviation
